# Cytogenetic and molecular predictors of response in patients with myeloid malignancies without del[5q] treated with lenalidomide

**DOI:** 10.1186/1756-8722-5-4

**Published:** 2012-03-05

**Authors:** Yuka Sugimoto, Mikkael A Sekeres, Hideki Makishima, Fabiola Traina, Valeria Visconte, Anna Jankowska, Andres Jerez, Hadrian Szpurka, Christine L O'Keefe, Kathryn Guinta, Manuel Afable, Ramon Tiu, Kathy L McGraw, Alan F List, Jaroslaw Maciejewski

**Affiliations:** 1 Department of Translational Hematology and Oncology Research, Cleveland Clinic Taussig Cancer Institute, Cleveland, OH, USA; 2 Hematologic Oncology and Blood Disorders, Cleveland Clinic Taussig Cancer Institute, Cleveland, OH, USA; 3 H. Lee Moffitt Cancer Center, Tampa, FL, USA; 4 Translational Hematology and Oncology Research, Taussig Cancer Institute R-40, Cleveland Clinic, 9500 Euclid Ave, Cleveland, OH, USA

**Keywords:** Lenalidomide, del[5q], Metaphase cytogenetics, Fluorescence in situ hybridization, Single nucleotide polymorphism array

## Abstract

**Background:**

While lenalidomide (LEN) shows high efficacy in myelodysplastic syndromes (MDS) with del[5q], responses can be also seen in patients presenting without del[5q]. We hypothesized that improved detection of chromosomal abnormalities with new karyotyping tools may better predict response to LEN.

**Design and methods:**

We have studied clinical, molecular and cytogenetic features of 42 patients with MDS, myeloproliferative neoplasms (MPN), MDS/MPN overlap syndromes and secondary acute myeloid leukemia (sAML) without del[5q] by metaphase cytogenetics (MC) who underwent therapy with LEN.

**Results:**

Fluorescence in situ hybridization (FISH) or single nucleotide polymorphism array (SNP-A)-based karyotyping marginally increased the diagnostic yield over MC, detecting 2/42 (4.8%) additional cases with del[5q], one of whom were responded to LEN. Responses were more often observed in patients with a normal karyotype by MC (60% vs abnormal MC; 17%, *p *= .08) and those with gain of chromosome 8 material by either of all 3 karyotyping methods (83% vs all other chromosomal abnormalities; 44% *p *= .11). However, 5 out of those 6 patients received combined LEN/AZA therapy and it may also suggest those with gain of chromosome 8 material respond well to AZA. The addition of FISH or SNP-A did not improve the predictive value of normal cytogenetics by MC. Mutational analysis of *TET2, UTX, CBL, EZH2, ASXL1, TP53, RAS, IDH1/2*, and *DNMT-3A *was performed on 21 of 41 patients, and revealed 13 mutations in 11 patients, but did not show any molecular markers of responsiveness to LEN.

**Conclusions:**

Normal karyotype and gain of chromosome 8 material was predictive of response to LEN in non-del[5q] patients with myeloid malignancies.

## Background

Lenalidomide (LEN) is particularly effective in patients with myelodysplastic syndromes (MDS) and the del[5q] cytogenetic abnormality [1-3]. In MDS-003, the phase II registration trial of 148 lower-risk MDS patients with del[5q] with or without other karyotypic abnormalities, 67% achieved transfusion independence with a complete and partial cytogenetic response rate of 45% and 28%, respectively [2]. There was no significant association between karyotypic complexity and the frequency of a cytogenetic response. LEN also has activity in a proportion of MDS without del[5q] [4] and [5]. Transfusion-dependent MDS patients low- or Int-1 by the International Prognostic Scoring System (IPSS) without del[5q] achieved a 43% overall rate of hematologic improvement [4]. However, there were no significant differences in the rate of transfusion independence according to age, sex, FAB type, IPSS category, cytogenetic pattern, or early cytopenias. In higher risk (IPSS; Int-2, high) MDS patients with del[5q] with or without other karyotypic abnormalities, 27% achieved complete remission (CR), including 67% of patients with isolated del[5q], vs. 1/1 and 0/27 patients with one or more than one additional chromosomal abnormalities, respectively (*P *< .001) [3]. Recently, high-dose LEN therapy resulted in a 14% CR/partial response (PR) rate in AML patients with del[5q] [6], and a 30% CR/complete remission without complete recovery of all blood counts (CRi) rate in older AML patients without del[5q] [5]. To date, the presence of del[5q] with or without additional chromosomal abnormalities detected by metaphase cytogenetics (MC) remains the best prognostic factor for response to LEN. As patients without del[5q] can also show responses to LEN, identification of additional markers of response/resistance is of utmost importance. Clinically, cytogenetic abnormalities including cryptic deletions of 5q, along with certain other mutations, may constitute additional lesions predictive of response. For instance, the presence of *TP53 *mutations has been shown to be associated with poor prognosis in azacitidine-treated MDS patients [7], and in LEN-treated MDS or AML patients with del[5q] [8,9].

The diagnostic yield of MC can be enhanced by application of fluorescence in situ hybridization (FISH) for targeted detection of chromosomal lesions including del[5q], as this technique is considered to be more sensitive and allow for detection of smaller clones [10]. Similarly, single nucleotide polymorphism array (SNP-A)-based karyotyping, due to its superb resolution, may allow for detection of previously cryptic unbalanced chromosomal defects [10] and [11]. Both techniques can be performed on interphase cells, and thereby do not require cell division.

In addition to mostly unbalanced cytogenetic defects, mutations of a number of genes, including *TET2 *[12,13], *UTX *[14], *CBL *[15], *EZH2 *[16-18], *ASXL1 *[19-21], *TP53 *[7,22,23], *RAS *[24,25], *IDH1/2 *[26], and *DNMT3A *[27] have been implicated in the pathogenesis of MDS and may also modulate clinical features including responsiveness to LEN.

We examined a cohort of patients without del[5q] treated with LEN and explored the relationship between molecular features and clinical response to LEN.

## Methods

### Patients

Bone marrow (BM) and/or peripheral blood (PB) were collected from 755 patients with myeloid malignancies seen at Cleveland Clinic (CC) and H. Lee Moffitt Cancer Center between 2002 and 2010. First, a cohort of 122 patients, who were examined with all 3 cytogenetic methods (MC, FISH and SNP-A) on the same sample, was collected. Next, data from 42 patients with MDS (31; RA, 5; RARS, 13; RCMD, 1; RAEB-1, 4; RAEB-2, 7; MDS-U, 1), myeloproliferative neoplasms (MPN) (PMF, 2), MDS/MPN overlap syndrome (7; CMML-1, 2; CMML-2, 2; MDS/MPN-U, 3), or 2 secondary acute myeloid leukemia (sAML) without del[5q], who received LEN for at least 8 weeks, were collected retrospectively. The schedule and dosage of lenalidomide was primarily 10 mg/day (5 mg/day in a few cases) for 1-21 days, with a 28-day cycle. All bone marrow biopsies were reviewed and diagnoses confirmed at Cleveland Clinic and H. Lee Moffitt Cancer Center. Response to LEN was defined by the modified International Working Group (IWG) response criteria (2006) [28]. Informed consent for sample and clinical information collection was obtained according to protocols approved by the Cleveland Clinic or the H. Lee Moffitt Cancer Center IRBs.

### Cytogenetic analysis

**Figure 1:**
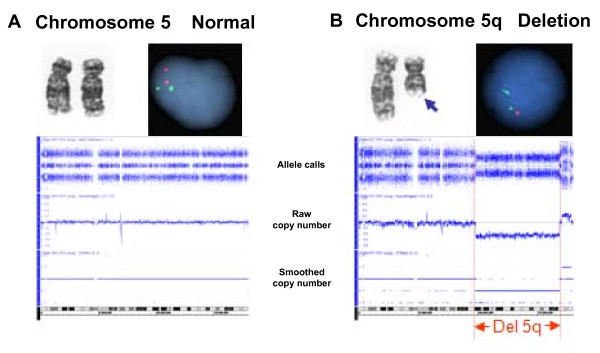
**Detection of chromosome 5 abnormalities by different cytogenetic techniques**. Examples of normal chromosome 5 (A) and deleted chromosome 5 (B) are presented. The deleted lesion is denoted by a shorter chromosome in MC (top left panel, blue arrow), a single red signal in FISH (top right panel) and segmental copy number loss in the SNP-A karyotype (bottom panel).

Cytogenetic analysis was performed on marrow aspirates and/or peripheral blood, in cases where bone marrow samples could not be obtained, according to standard methods (Figure 1). 20 metaphase spreads were examined per patient, if available. Chromosome preparations were G-banded using trypsin and Giemsa (GTG) and karyotypes were described according to ISCN [29].

### Fluorescence in situ hybridization

FISH analysis was performed on cell pellets from unstimulated cytogenetic cultures. Thresholds for interpretation as a positive result were established for each probe at 3 standard deviations above the mean of 20 normal bone marrow samples. In 27 cases, FISH analysis was performed at an outside reference laboratory (Mayo Clinic) using the following dual color probe sets: 5p15.2 (normal range; 0-4%)/EGR1 (5q31) (0-6%), CEP7 (0-5%)/7q31 (0-7%), CEP8 (0-2%)/MYC (8q24) (0-2%) and 20q12 (0-5%)/20qter (0-5%). In 95 cases, FISH was performed at CC using three dual color probe sets (Abbott Molecular, Abbott Park, IL). The first probe set consisted of D5S23 and D5S721 (5p15.2) labeled in Spectrum Green (0-6%) and EGR1 (5q31) labeled in Spectrum Orange (0-6%). The second probe set consisted of the chromosome 7 centromere labeled in Spectrum Green (0-5%) and D7S486 (7q31) labeled in Spectrum Orange (0-7%). The third probe set consisted of the chromosome 8 centromere labeled in Spectrum Green (0-8%) and D20S108 (20q12) labeled in Spectrum Orange (0-4%).

### DNA extraction

DNA was extracted from whole bone marrow using the ArchivePure Kit (5Prime, Gaithersburg, MD) per manufacturer's instructions. The concentration of the DNA was obtained using a ND-1000 spectrophotometer (NanoDrop, Wilmington, DE, USA). To study the germ line, T lymphocytes (CD3+) were isolated using RoboSep according to manufacturer's protocol (StemCell Technologies, Vancouver, BC, Canada).

### Mutational analysis

Mutation screening was performed for genes known to be mutated in myeloid malignancies (*TET2, UTX, CBL, EZH2, ASXL1, TP53, RAS, IDH1/2*, and *DNMT3A*) in the cases for which DNA was available (N = 21). All of the coding exons for *TET2, UTX, EZH2 *and *TP53 *were screened as previously reported [13-17] and [23]. Direct genomic sequencing of exons 8 and 9 of *CBL*, exon 12 of *ASXL-1*, exons 1 and 2 of *N-RAS *and *K-RAS*, exon 4 of *IDH-1 *and *IDH-2*, and exon 23 of *DNMT3A *was performed as previously described [15,21,26,27,30]. The reference sequence from UCSC Genome Browser was used to identify the position of each amino acid change listed in Table 1 (*TET2*, uc003hxk.2; *EZH2*, uc003wfb.1; *ASXL-1*, uc002wxs.1; *KRAS*, uc001rgp.1; *DNMT-3*, uc002rgc.1). In selected cases CD3+ cells were purified and used as controls to confirm the somatic status of mutations.

**Table 1:** Mutation analysis in the cohort of LEN patients

Case	Response	*TET2*	*UTX*	*CBL*	*EZH2*	*ASXL1*	*TP53*	*N-RAS*	*K-RAS*	*IDH1*	*IDH2*	*DNMT3A*
1	CR	WT	WT	WT	WT	WT	WT	WT	WT	WT	WT	WT
2	CR	WT	WT	WT	WT	L775fsX1	WT	WT	WT	WT	WT	WT
3	PR	WT	WT	WT	WT	WT	WT	WT	WT	WT	WT	WT
4	NR	WT	WT	WT	WT	WT	WT	WT	WT	WT	WT	WT
5	CR	WT	WT	WT	WT	E1102D	WT	WT	WT	WT	WT	R882H
6	PR	P1681fsX2	WT	WT	WT	P1277fsX2	WT	WT	WT	WT	WT	WT
7	CR	WT	WT	WT	WT	WT	WT	WT	WT	WT	WT	WT
8	HI	WT	WT	WT	WT	WT	WT	WT	WT	WT	WT	WT
10	CR	WT	WT	WT	WT	WT	WT	WT	WT	WT	WT	WT
11	HI	WT	WT	WT	WT	WT	WT	WT	L19F	WT	WT	WT
12	CR	V1417F	WT	WT	WT	WT	WT	WT	WT	WT	WT	WT
13	NR	WT	WT	WT	WT	WT	WT	WT	WT	WT	WT	WT
14	PR	WT	WT	WT	WT	WT	WT	WT	WT	WT	WT	WT
15	CR	T1978P	WT	WT	WT	WT	WT	WT	WT	WT	WT	WT
16	NR	WT	WT	WT	WT	S846N	WT	WT	WT	WT	WT	WT
17	NR	N1068fsX13	WT	WT	WT	WT	WT	WT	WT	WT	WT	WT
18	NR	WT	WT	WT	WT	WT	WT	WT	WT	WT	WT	WT
19	NR	P1962L	WT	WT	WT	WT	WT	WT	WT	WT	WT	WT
20	CR	WT	WT	WT	WT	WT	WT	WT	WT	WT	WT	R882H
21	HI	WT	WT	WT	T726X	WT	WT	WT	WT	WT	WT	WT
22	HI	WT	WT	WT	WT	WT	WT	WT	WT	WT	WT	WT

Abbreviation: *CR *complete response; *PR *partial response; *HI *hematologic improvement; *WT *wild type

### SNP-A cytogenetics

Affymetrix Gene Chip Mapping 250 K Assay Kit or Genome-Wide Human SNP Assay Kit 6.0 (Affymetrix, Santa Clara, CA) was used for analysis of 52 and 70 samples with myeloid malignancies, respectively. Following Nsp I digestion (New England Biolabs, Ipswich, MA), fragmented DNA was ligated to adaptor using T4 ligase (New England Biolabs) followed by PCR amplification. The PCR product was hybridized to the array, processed with the Fluidic Station 450 and scanned using the Gene Chip Scanner 3000 (Affymetrix).

### Biostatistical evaluation of SNP-A data

GeneChip Mapping 250 K Array data, signal intensity and SNP calls were determined using Gene Chip Genotyping Analysis Software Version 4.0 (GTYPE). Copy number and LOH were investigated using Copy Number Analyzer for Affymetrix GeneChip Mapping (CNAG v. 3.0). For Genome-Wide Human SNP Array 6.0, the genotype calls for each individual were determined by the Birdseed version 1 genotype-calling algorithm, embedded in the software included with the Affymetrix Genotyping Console 2.0 (Affymetrix).

For detection of lesions we used the following diagnostic algorithm: lesions identified by SNP-A were compared with the Database of Genomic Variants (http://projects.tcag.ca/variation/) and our own internal control series to exclude known copy number variants (CNVs). In our internal control cohort, the largest area of copy neutral loss of heterozygosity (CN-LOH) we observed was 52.5 Mb and the average size of CN-LOH was 7.2 Mb. In addition, we observed that areas of LOH in controls were exclusively interstitial. Consequently, areas of LOH < 24.8 Mb (mean size ± 2SD) were excluded from analysis in the patient set. Deletions and gains of chromosomal material seen on metaphase karyograms and SNP-A samples that showed a concordantly normal karyotype by both MC and SNP-A testing were not further confirmed. When possible, all other remaining new defects were confirmed using paired analysis of CD3+ cells.

### Statistical analysis

Demographic and baseline MDS disease characteristics of all patients were summarized descriptively, using medians and ranges. The response differences between 2 groups were compared using Fisher's exact test, with a two-sided alpha value of .05 denoting significance.

## Results

### Comparison between metaphase cytogenetics and other cytogenetic methods

We first identified a cohort of 122 patients for whom MC, FISH and SNP-A analyses were performed on the same sample, to evaluate the additional yield of more sensitive techniques for identifying del[5q]. In patients with MDS (N = 82), MDS/MPN (N = 13), AML (N = 23), and MPN (N = 4), the detection rate of del[5q] increased only marginally with the use of additional techniques, from 24% (MC + FISH), to 25% (MC + SNP-A), 25% (FISH + SNP-A) and 26% (all 3 methods) (Figure 1, Table 2). We also identified 3 cases with copy neutral loss of heterozygosity (CN-LOH) of 5q in this cohort using SNP-A. One region of CN-LOH was found in sAML with complex chromosomal abnormalities including del[5][q13q33] by MC, and the other 2 CN-LOH regions were detected in MDS cases with chromosomal abnormalities other than del[5q].

**Table 2:** Number and percentage of del[5q] detected using metaphase cytogenetics, FISH and SNP-A alone or in combination in myeloid malignancies (N = 122)

	MC	FISH	SNP	MC+FISH	MC+SNP	FISH+SNP	MC+FISH+SNP
Number	24	27	25	29	30	31	32
Percentage	20%	22%	21%	24%	25%	25%	26%

*MC *metaphase cytogenetics; *FISH *fluorescence in situ hybridization; *SNP *single nucleotide polymorphism array-based karyotyping.

### Clinical characteristics of non-del[5q] patients who had LEN therapy

Clinical characteristics of the patients with myeloid malignancies without del[5q] by MC and who received LEN are summarized in Table 3; 31 patients received LEN monotherapy (complete response [CR], N = 3; partial response [PR], N = 2; hematologic improvement [HI], N = 9; no response [NR], n = 13; not evaluated [NE], n = 4), and 11 patients received LEN/azacitidine (AZA) combination therapy (CR, N = 6; PR, N = 1; HI, N = 1; NR, N = 3). Only 1 patient had past history of Hodgkin's lymphoma and was suspected to have therapy-related MDS/MPN.

**Table 3:** Summary of clinical characteristics of patients without del[5q] on MC who received lenalidomide (N = 42)

Diagnosis (No. of Patients)			
MDS		31	
	RA		5
	RARS		13
	RCMD		1
	RAEB-1		4
	RAEB-2		7
	MDS-U		1
MDS/MPN		7	
	CMML-1		2
	CMML-2		2
	MDS/MPN-U		3
PMF		2	
sAML		2	

Age (years old)			

	Median (Range)		70 (46-83)

Sex (No. of Patients)			

	M		28
	F		14

IPSS (No. of Patients)			

	LOW		12
	INT-1		11
	INT-2		7
	HIGH		1
	not indicated		11

Duration of MDS (months)			

	Median (Range)		15 (0-118)

Previous Therapies			

	Yes		27
	No		15

Transfusion dependence (No. of Patients)			

	Yes		30
	No		12

Neutropenia (< 1.5 × 10^9^/μ) (No. of Patients)			

	Yes		5
	No		37

Thrombocytopenia (< 100 × 10^9^) (No. of Patients)			
	Yes		12
	No		30

Therapy (No. of Patients)			

LEN (5-10 mg/day) alone			30
LEN high dose (50 mg/day)			1
	LEN/AZA		11

Duration of LEN therapy (months)			

	Median (Range)		5 (0-76)

Response to therapy (No. of Patients)			

	CR		9
	PR		3
	HI		10
	NR		16
	NE		4

Abbreviation: *MDS *myelodysplastic syndromes; *RA *refractory anemia; *RARS *refractory anemia with ring sideroblasts; *RCMD *refractory cytopenia with multilineage dysplasia; *RAEB *refractory anemia with excess blasts, *MDS-U *MDS unclassifiable; *MDS/MPN *MDS/myeloproliferative neoplasm; *CMML *chronic myelomonocytic leukemia; *MDS/MPN-U *MDS/MPN unclassifiable; *PMF *primary myelofibrosis; *sAML *secondary acute myeloid leukemia; *M *male; *F *female; *LEN *lenalidomide; *LEN/AZA *LEN/azacitidine; *CR *complete response; *PR *partial response; *HI *hematological improvement; *NR *no response; *NE *not evaluated.

**Figure 2:**
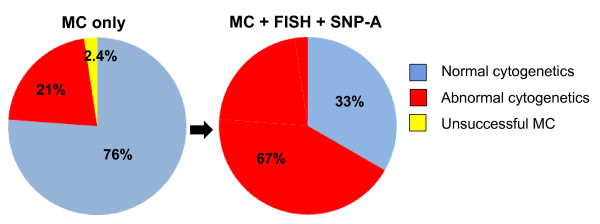
**Frequency of cytogenetic abnormalities by MC only, or by MC, FISH, and SNP-A**. Compared to MC only (left), addition of FISH and SNP-A (right) improved the detection rate of chromosomal abnormalities dramatically from 21% to 67% in patients receiving LEN without del(5q) by MC (N = 42).

By MC, 32 patients (76%) who received treatment with LEN showed a normal karyotype, 1 patient (2.4%) had no growth to their bone marrow sample, and 9 (21%) had an abnormal karyotype other than del[5q] (Table 4). However, the frequency of an abnormal karyotype was increased to 67% using FISH and SNP-A as karyotyping tools in patients receiving LEN without del[5q] by MC (Figure 2). Previously cryptic del[5q] was detected by both SNP-A and FISH in an additional 1/18 patients with normal MC (Case 19 in Table 4). Del[5q] was also revealed by FISH in 1 patient with unsuccessful MC (Case 14 in Table 4), but, due to the small size of the clone (8%), SNP-A was not able to detect this lesion.

**Table 4:** Patients characteristics who received LEN without del[5q] by MC (N = 42)

Case	Age (y.o.)	Sex	Diagnosis	IPSS	Therapy	Response	MC	FISH	SNP-A		
1	73	M	RAEB-2	INT-2	LEN/AZA	CR	N	N		Gain	4q13.2
2	75	M	RAEB-1	INT-1	LEN/AZA	CR	N	trisomy 8	9%	Gain	8q11.1q11.21
										UPD	11q14.1q21
3	62	M	MDS/MP N-U	INT-1	LEN/AZA	PR	N	N		UPD	1pterp32.3
										UPD	3p21.31p21.1
4	68	M	RAEB-2	HIGH	LEN/AZA	NR	complex karyotype, including trisomy 8, del[7q], del[12], and del[20]	del[7q]	68%	Gain	8
								del[20q]	60%	Loss	11p14.3p13
								trisomy 8	41%	Loss	12p12.3p11.21
										Loss	12q21.1q21.31
										Loss	16q22.3q24.3
										Loss	1p22.2p22.1
										Loss	20q11.2q13.33
										Loss	21q11.2q21.1
										Loss	2q31.3q32.1
										Loss	6q23.3
										Gain	7q11.21q11.22
										Loss	7q22.1q36.2
5	68	M	RAEB-2	INT-2	LEN/AZA	CR	47,XY,+8[6]	trisomy 8	9%	Gain	14q11.1q11.2
										UPD	19p13.11p12
										Gain	8p23.3q24.3
6	73	M	CMML-1	NE	LEN	PR	N	del[20q]	10%	UPD	9pterp22.2
7	67	M	PMF	NE	LEN	PR	N	N		Loss	11q23.3
8	66	M	RCMD	LOW	LEN	HI	46,XY,del(20)(q11q13)[2]/4 6,XY[18]	del[20q]	17%	Gain	8q11.1q11.23
9	69	M	RAEB-1	INT-2	LEN/AZA	CR	N	N		NE	
10	64	M	MDS/MP N-U	NE	LEN	CR	N	N		N	
11	79	F	CMML-2	NE	LEN/AZA	HI	47,XX,+8[20]	trisomy 8	42%	Gain	8
12	62	F	RAEB-2	INT-2	LEN/AZA	CR	N	N		N	
13	62	F	RARS	INT-1	LEN	NR	46,XX,add(15)(p11.1),add(2 2)(p11.2)[3]/47,idem,+19[19].	del[7q]	11%	Gain	19
								del[7]	6%	Gain	3q26.1
										Gain	4p16.2
14	72	M	MDS/MPN-U	INT-2	LEN	PR	no growth	del[5q]	8%	Loss	20q11.1q13.12
								del[20q]	35%	Loss	2p21p24.1
										Loss	8q11.23q12.1
										Gain	9p12pter
15	63	M	RAEB-2	NE	LEN/AZA	CR	N	N		Gain	12q24.32
										Gain	8q11.1
16	81	F	RAEB-2	INT-2	LEN/AZA	NR	N	N		N	
17	69	M	CMML-2	-	LEN/AZA	NR	N	NE		Loss	2p22.3
18	46	M	PMF	-	LEN	NR	balanced translocation at chromosomes 2 and 22	NE		UPD	14q31.3q32.33
										Gain	9p24.3p11.1
19	70	F	sAML	-	LEN (High dose)	NR	N	del[5q]	33%	Loss	5q31.2
20	65	F	RARS	LOW	LEN	CR	N	NE		N	
21	70	M	CMML-1	INT-1	LEN	HI	N	N		UPD	7q22.1qter
22	83	M	RARS	LOW	LEN	HI	N	N		N	
23	83	M	RARS	INT-1	LEN	NR	N	NE		N	
24	71	M	MDS-U	INT-1	LEN	NE	N	NE		Loss	15q14
25	69	M	RARS	LOW	LEN	NR	N	NE		N	
26	76	M	RA	INT-1	LEN	NR	N	NE		Loss	3p22.3
27	68	M	RA	INT-1	LEN	NR	N	NE		Loss	21q21.2
										Gain	3p14.1
										Loss	11q14.3
28	59	M	RARS	LOW	LEN	NR	N	NE		N	
29	73	F	RA	LOW	LEN	NR	N	NE		N	
30	78	F	RARS	LOW	LEN	NE	N	NE		UPD	3q21.3qter
31	78	M	RAEB-1	INT-2	LEN	NR	47,XY,+19	NE		Gain	19
32	80	M	RAEB-1	INT-1	LEN	NE	N	NE		Loss	17q11.2
										UPD	8p11.2qter
33	79	F	RARS	INT-1	LEN	HI	N	NE		N	
34	73	F	RARS	LOW	LEN	HI	N	NE		Loss	18p11.32
35	78	F	sAML	-	LEN	NR	46,XX,t(3;3)	NE		Loss	3q26.1
36	80	M	RAEB-2	INT-2	LEN	NR	N	NE		Loss	22q13.2
37	69	M	RARS	LOW	LEN	HI	N	NE		N	
38	59	F	RA	LOW	LEN	HI	N	NE		N	
39	62	F	RARS	LOW	LEN	NE	N	NE		Gain	6p21.32
40	82	M	RARS	INT-1	LEN	NR	46,XY,del(20)(q11.2)	NE		Loss	20q11.2q13.2
41	53	M	RARS	LOW	LEN	HI	N	NE	N		
42	56	F	RA	INT-1	LEN	HI	N	NE		Loss	9p21.2
										Gain	1p21.1

Abbreviation: *M *male; *F *female; *RAEB *refractory anemia with excess blasts; *MDS/MPN-U *myelodysplastic syndromes/myeloproliferative neoplasm, unclassifiable; *CMML *chronic myelomonocytic leukemia; *PMF *primary myelofibrosis; *RCMD *refractory cytopenias with multilineage dysplasia; *RARS *refractory anemia with ring sideroblasts; *sAML *secondary acute myeloid leukemia; *MDS-U*, MDS unclassifiable; *RA *refractory anemia; *LEN *lenalidomide; *AZA *azacitidine; *N *normal; *UPD *uniparental disomy; *NE *not evaluated.

### Impact of chromosomal abnormalities on response

In 27 patients who received LEN for more than 2 months, the overall response rate (ORR) was 52%, including 3 CR, 2 PR and 9 HI. Case 19, who was diagnosed as sAML with del[5q] by FISH and SNP-A only, was refractory to high-dose LEN. Case 14, a MDS/MPN unclassifiable (MDS/MPN-U) patient in whom a del[5q] clone was detected only by FISH due to the small size, had a sustained PR with transfusion independence. The ORR to LEN in patients with normal MC was 60%, vs. 17% for those with chromosomal aberrations by MC (*p *= .08); the addition of FISH or SNP-A did not improve the predictive value of normal cytogenetics (Table 5). We also analyzed 11 patients without del[5q] by MC who received combination therapy with AZA and LEN, for whom the ORR was 73% (6 CR, 1 PR, 1 HI). By MC, 8/11 patients had a normal karyogram and a response of 75%, compared to 3 patients with chromosomal lesions, 1 of whom did not respond. Similar to the results with LEN alone, inclusion of defects detected by SNP-A or FISH did not allow for better separation of responders based on normal cytogenetics by MC. The most frequent cytogenetic abnormality among the patients who received LEN was gain of chromosome 8 material (6/42), followed by the loss of chromosome 20 material (5/42). Patients with gain of chromosome 8 had high ORR to LEN (5 out of 6, 83%) and ORR in patients with chromosome 20 abnormalities was 3 out of 5. The response of patients with all other chromosomal abnormalities by MC, FISH or SNP-A was 44%. These findings indicate that responses tend to be more often observed in patients with gain of chromosome 8 material by either of all 3 karyotyping methods (*p *= .11), although 5 out of those 6 patients received combined LEN/AZA therapy.

**Table 5:** Cytogenetic categories and response to therapy in the cohort of LEN patients

A. All patient who received LEN for more than 3 months (N = 38)			
	Normal cytogenetic group	Abnormal cytogenetic group	p value
Categorized by MC only	64%	33%	0.07
Categorized by MC/FISH/SNP-A	64%	54%	0.4

B. Monotherapy (LEN only) patients (N = 27)			
	Normal cytogenetic group	Abnormal cytogenetic group	p value

Categorized by MC	60%	17%	0.08
only			
Categorized by MC/FISH/SNP-A	64%	44%	0.27
C. Patients with combination therapy of AZA + LEN (N = 11)			

	Normal cytogenetic group	Abnormal cytogenetic group	p value

Categorized by MC only	75%	67%	0.85
Categorized by MC/FISH/SNP-A	67%	75%	0.85

*LEN *lenalidomide; *MC *metaphase cytogenetics; *FISH *fluorescence in situ hybridization; *SNP-A *single nucleotide polymorphism array karyotyping; *AZA *azacitidine.

### Impact of mutational status on response

In 21 LEN-treated patients (11 patients with LEN only and 10 patients with LEN/AZA), somatic mutations were found in *TET2 *(N = 5), *EZH2 *(N = 1), *ASXL1 *(N = 4), *K-RAS *(N = 1), and *DNMT3A *(N = 2) in 11 patients. *ASXL1 *and *DNMT3*, or *TET2 *and *ASXL1 *mutations were each found in one patient, and each of these patients achieved CR with LEN/AZA or PR with LEN only. ORR was 73% in patients with any of investigated mutations and 70% in patients without a mutation (*p *= .36). For patients treated with LEN only, 3 out of 8 (38%) responders had mutations, and 1 out of 3 (33%) non-responders harbored mutations.

## Discussion

Though the mechanism of action of lenalidomide has not been definitively determined, it purportedly works through inhibition of phosphatase activity in the common deleted region (CDR) of 5q that plays a key role in cell cycle regulation, through a defect in ribosomal protein function, via direct cytotoxic mechanisms in patients with the del (5q) cytogenetic abnormality, and supposedly through effects on the bone marrow microenvironment in patients who do not have this lesion, via abrogation of the effects of pro-apoptotic, pro-inflammatory cytokines [1-3]. Until now, additional markers of responsiveness to LEN beyond del[5q] have not been identified.

New cytogenetic tools such as FISH and SNP-A are likely to improve the diagnostic value of cytogenetic diagnostics [8,9,29]. We first assumed that we would detect previously unrecognized cases of del[5q] using these techniques. In our cohort of patients given LEN, del[5q] cases detected by FISH and/or SNP-A ranged from 2 out of all 42 cases (4.8%) of patients without del[5q] by MC, and 1 out of 32 cases (3.1%) with normal MC. These frequencies are similar to those reported in a previous study, in which 5.96% of cases without del[5q] by MC and 2.7% of those with normal karyotype by MC were found to be del[5q] by FISH [31]. These results suggest that FISH and SNP-A may marginally improve the detection rate of del[5q]. While the detection rate of cryptic del[5q] was only marginally enhanced with FISH and SNP-A, new karyotyping tools improved the detection rate of other chromosomal abnormalities in our cohort from 21% to 67% compared to MC. A previous study of 43 MDS patients suggested that the cytogenetic pattern correlates with hematologic response; 10 of 12 patients (83%) with del[5q] achieved sustained red blood cell transfusion independence, compared with 57% of those with a normal karyotype and 12% of those with other karyotypic abnormalities [1,32]. Consequently, we hypothesized that improved detection of chromosomal abnormalities may better predict poor response to LEN. However, when more abnormalities were found using additional methods, no correlation with response to LEN or LEN/AZA therapy was detected.

We also speculated that we could recognize other chromosomal markers of response or refractoriness to LEN besides del[5q] using FISH and SNP-A. For example, trisomy 13 as the sole cytogenetic abnormality was reported to be possible good prognostic factor to LEN therapy [33], but was not detected in our LEN cohort. Instead, we found gain of chromosome 8 material to be predictive of response to LEN, although we acknowledge that 5 out of those 6 patients received combined LEN/AZA therapy [34,35].

In addition to cytogenetic abnormalities, we also studied mutational status of a variety of genes as possible markers of response. For example, *ASXL1 *mutations in CMML [36] and *DNMT3A *in AML [27] were reported to be poor prognostic factors. We identified 2 patients with *DNMT3A *mutations in our cohort, both of them achieved CR with LEN or LEN/AZA therapy.

This cohort used in this study has several limitations, including a limited size and the inclusion of patients with heterogenous disease entities. But, we have been able to demonstrate that a normal karyotype and gain of chromosome 8 material was predictive of response to LEN, while additional testing by FISH or SNP-A is not useful for better prediction of response in non-del[5q] patients with myeloid malignancies.

## Competing interests

The authors declare that they have no competing interests.

## Authors' contributions

YS and JPM designed research, performed research, analyzed data, and wrote the paper. HM, FT, VV, AJ, AJ, HS, CLO, KG performed research. MA, RT, KRM and AFL analyzed data. All authors read and approved the final manuscript.
